# Supplementary material for: Are carbon emissions trading and green financial instruments synergistic? -Comprehensive quantitative research based on content analysis
Source: PLoS One. 2024 Mar 7;19(3):e0298601. doi: 10.1371/journal.pone.0298601 (PMC10919601; doi:10.1371/journal.pone.0298601)
Supplement: S1 Data — (ZIP) [file pone.0298601.s001.zip › Data/地级市Z_F工作报告.zip!/╔╜╢1⁄2/╫═▓⌐╩╨/└┤╫╘╣1⁄2╓┌║┼í╛┬φ┐╦╩2╛▌═°í┐_2012.docx]

各位代表：

现在，我代表市人民政府，向大会作政府工作报告，请予审议，并请各位政协委员和其他列席会议的同志提出意见。

一、过去四年政府工作回顾

过去四年是我市发展史上极不平凡的四年。在中共淄博市委的坚强领导下，全市上下深入贯彻落实科学发展观，积极应对国际金融危机等各种风险挑战，坚定不移地走内涵发展的路子，在顺利完成“十一五”规划的基础上，提前一年实现了市十三届人大一次会议确定的主要目标任务，谱写了老工业城市科学发展的新篇章。

——这是全市综合实力显著增强的四年。2011年，全市工业总产值和主营业务收入比2007年实现翻番，双双突破1万亿元，我市成为全省第3个、全国第16个工业经济过万亿的城市；全市地区生产总值实现3280.2亿元，是2007年的1.63倍；境内财政总收入达到476.2亿元，其中地方财政收入突破200亿元，是2007年的2.08倍；金融机构人民币存款余额达到2725亿元、贷款余额达到1872亿元，分别是2007年的1.95倍和1.94倍；城市居民人均可支配收入达到24955元，农民人均纯收入达到10878元，分别是2007年的1.57倍和1.68倍。全市综合实力跨上了一个大台阶。四年来，面对历史罕见的国际金融危机冲击和极其复杂的国内外环境，我们坚持咬定发展不放松，始终把保持经济平稳较快增长紧紧抓在手上。注重把保发展与增后劲结合起来，抓住机遇，策划实施了“两区三村”改造建设工程、新型城镇化以及传统产业技改计划等一大批重点项目，既拉动了即期增长，又为长远发展积蓄了力量，四年累计完成固定资产投资4608亿元，是上一个五年的2.08倍。同时，注重促进投资、消费、出口协调拉动，全市经济自主增长、内生增长的动力明显增强，固定资产投资、社会消费品零售总额、外贸进出口总额年均分别增长24.05%、19.63%和17.86%。四年来，我们坚持务实保发展与增创新优势“两手抓”，在经济总量跃上新水平的同时，在全国资源型老工业城市中率先创建为全国文明城市，创造性地设立齐鲁股权托管交易中心、淄博保税物流中心等重大资本和开放平台，淄博的比较优势加速放大，城市地位和影响力显著提升。

——这是经济结构调整取得重大进展的四年。四年来，我们坚持把推进经济结构的战略性调整，作为老工业城市转型升级的主攻方向。持续组织实施了一大批“工业百项”重点项目，累计完成技改投资2093亿元，一批骨干企业跃居行业领军地位，氯碱离子膜、超级动力电容电池等一批重大高新技术项目实现产业化，新材料、精细化工等新兴产业竞争力日益增强。中心城区东部化工区以及张钢片区搬迁改造、南部区域综合整治取得重大进展，产业布局调整大见成效。全市工业整体素质和效益明显提升，2011年，全市主营业务收入过百亿的企业达到10家，省级以上企业研发机构达到209家，中国名牌产品、中国驰名商标总数分别达到30个和47件，均居全省前列。我们着力引进优质资本和品牌，高水平策划、高效能推进了文昌湖省级旅游度假区、华润城市综合体、淄博太空港航天科技文化城、太保电话保险运营中心等一批高端服务业项目，全市现代服务业发展进入提质增效、带动经济结构升级和城市品质提升的新阶段。全市粮食生产连续九年增收，有机农业、品牌农业、都市农业发展势头良好，“三品一标”产品达到238个，市级以上农业龙头企业达到122家，农业综合效益逐年提高。2011年，全市三次产业比例调整为3.6∶60.2∶36.2，四年间三产比重提高4.6个百分点，高新技术产业产值占比达到26.6%(新口径)，淄博老工业城市新的竞争优势正在加速形成。

——这是新型城镇化快速推进的四年。全市城镇化率超过64%，比2007年提高2.9个百分点，比全国、全省平均水平高出10个百分点以上，城乡规划建设管理水平显著提高。四年来，中心城区新城区建设有序展开，旧城区改造提升力度加大，相继建成市体育中心、运动员公寓、淄博客运中心等一批标志性建筑，开工建设市文化中心和新城区景观水系，大力推进城区道路、管网改造和旧居住区综合整治，全线贯通中心城区大外环，现代化城市的发展框架进一步拉开，城市形象与功能不断提升。与此同时，区县域城乡建设和生态建设、民生建设亮点纷呈，“四个层级”协调发展的格局进一步形成，“建设农村殷实小康十大工程”向纵深推进。今年上半年，历时逾三年、惠及城乡15万户居民的“两区三村”改造建设工程将基本完工。四年新建改造农村公路1564公里，农村自来水普及率达到97.6%，镇村面貌发生重大变化。在过去的四年中，我们组织实施了一批重大基础设施项目，大中小型水库除险加固工程全面完成，泰青威天然气管线淄博段在全省率先竣工输气，引太入张工程竣工投用，晋豫鲁铁路沂源段、南水北调淄博段进展顺利，累计改造、新建干线公路268公里，城市承载和辐射带动功能明显增强。

——这是生态文明建设水平显著提高的四年。四年间，全市万元GDP能耗累计下降21.36%，化学需氧量和二氧化硫排放量分别减少17.19%、27.87%，城市污水集中处理率达到93.5%，城市生活垃圾无害化处理实现全覆盖，主要河流水质全部达到恢复鱼类生长的要求，空气质量良好率超过50%、比2007年提高43.6个百分点。我市连续三年获得全省节能目标责任考核第一名，环保工作受到国家环保部表彰。作为一个结构性矛盾突出的老工业城市，能够取得这样的进展，实属不易，全市上下为此付出了前所未有的努力。四年来，我们强力推进节能减排，实施并完成新一轮“碧水蓝天行动计划”，新改扩建17座城镇污水处理厂，新配套污水管网300多公里，整治污染企业4800多家，关停各类“土小”企业3000多家，淘汰水泥落后产能1100万吨、钢铁落后产能437万吨、建陶落后产能9150万平方米。我们大力推进生态市建设，四宝山、孝妇河、猪龙河等一批重点生态修复工程取得显著成效，秸秆全面禁烧及转化利用工作成果不断扩大。全面提高大环境绿化水平，四年新增造林面积50万亩、园林绿地1200公顷，森林覆盖率达到36%，建成区绿化覆盖率达到42.6%，成功创建为全国绿化模范城市。人民群众热切期盼的让淄博天更蓝、地更绿、水更清、气更爽的目标正在加快变为现实。同时，我们更加清醒地认识到，淄博的生态文明建设任重而道远，需要全市上下为之长期艰苦奋斗！

——这是改革开放向纵深推进的四年。四年来，我们顺利完成了政府机构改革和乡镇布局调整，扎实推进马桥镇、凤凰镇、昆仑镇扩权强镇发展改革试点，加大对欠发达县、老工业区和强区县科学发展的支持与激励力度，区县域发展战略日益完善。先后争取高青县列入黄河三角洲高效生态经济区、沂源县享受国家支持沂蒙革命老区政策、淄川区列入国家资源枯竭型城市。我们着力深化企业改革，创造性地组织实施一系列重大资产重组，全部解决了上市公司历史遗留问题，同时新增上市公司9家，累计实现融资与再融资175亿元，资本市场上的“淄博板块”健康发展壮大。我们大力推进医药卫生体制改革，国家基本药物制度实现全覆盖，基层医疗卫生机构综合改革全面完成。集体林权制度改革成效显著，事业单位分类改革稳步启动，其他各项改革取得积极进展，经济社会发展活力不断增强。四年来，我们坚定不移地扩大对外开放，一批国内外知名企业入驻淄博，现代制造业和服务业等重点领域开放水平显著提高，全市累计实际利用外资22.9亿美元，是上一个五年的1.19倍。外贸发展方式加快转变，2011年，实现外贸进出口总额90.6亿美元，其中出口53.3亿美元，分别是2007年的1.93倍和1.78倍。以宏达矿业收购秘鲁邦沟铁矿、石嘴山淄博工业园建设为代表，“走出去”取得重大突破。我们成功举办了四届陶博会和新材料技术论坛，“新材料名都”和“淄博陶瓷·当代国窑”品牌影响力日益扩大。援川工作走在全省前列，援藏、援疆工作不断取得新成果。

——这是民生和社会建设显著加强的四年。坚持把保障和改善民生作为一切工作的出发点和落脚点，2008年至2011年，各级财政用于民生建设的支出累计达到398亿元，占财政总支出的52.5%，各项民生政策得到全面落实。四年来，我们认真落实各项就业和创业政策，累计新增城镇就业50.2万人次，农村劳动力转移就业33.6万人次，城镇登记失业率控制在3%以内。我们着力提高社会保障水平，城镇五项社会保险在全国同类城市中率先实现市级以上统筹，新农合、新农保和城镇居民养老、医疗保险实现全覆盖，惠及城乡全体居民的社会保障体系建设取得突破性进展。截至2011年末，全市社保基金积累103亿元，比四年前翻了一番，社会保障能力显著增强。妇女儿童、老龄、社会福利、慈善、残疾人、红十字等各项事业创新发展，社会救助体系日益完善。大力推进保障性安居工程建设，累计开工建设廉租房、公租房、经济适用房21965套，其中2011年开工建设12017套，超额完成省下达任务。

四年来，我们坚持教育优先发展战略，积极推进中小学校舍安全工程建设，新建中小学校17所，改造校舍面积150万平方米。科学整合职业教育资源，优化了教育资源配置。建立健全家庭困难学生资助体系，有效地促进了教育公平。2008年至2011年，全市地方财政预算内教育经费支出累计达169.55亿元，占地方财政支出总额的22.35%。四年来，我们先后完成投资5.34亿元，改造建设村卫生室1268处、县镇基层医疗卫生机构62处、市属医疗卫生机构10处，城乡医疗卫生保障能力显著增强。我市基本和重大公共卫生项目实施处于全国领先水平，卫生惠民力度不断加大。这四年，成为我市教育、卫生等社会事业投入力度最大、城乡办学和医疗条件改善最为明显的时期之一。我们大力加强精神文明建设，积极发展文化事业和文化产业，先后被评为全国未成年人思想道德建设先进市和全国文化体制改革先进地区。全市竞技体育和全民健身运动蓬勃发展，低生育水平保持稳定。四年来，我们积极创新兵员征集办法，扎实落实拥军优抚政策，实现全国双拥模范城“七连冠”。切实加强和创新社会管理，推进“法治淄博”建设，坚持以群众工作统揽信访工作，深入实施固本强基维稳工程和治安安民工程，总结推广“六小警务”经验，毫不放松地抓好安全生产和食品药品安全工作，建立健全应急管理体制机制，保持了社会大局和谐稳定，创建为全国社会治安综合治理优秀市和全国法制宣传教育先进市。民族宗教、外事、侨务、对台、防震减灾、气象、人防、统计、物价、史志、档案等各项事业都实现了新发展，取得了新成绩。

四年来，我们深入贯彻落实国务院《关于加强市县政府依法行政的决定》，扎实推进法治政府和服务型政府建设。不断完善行政决策机制，提高决策的科学化、民主化、法治化水平。自觉接受人大及其常委会的监督和政协的民主监督，虚心听取各民主党派、工商联和社会各界的意见，认真办理人大代表建议和政协委员提案。积极推进行政审批“两集中、两到位”改革，切实规范行政执法，加强政府信息公开和行政电子监察平台建设，努力提高行政效能。严格落实党风廉政建设责任制，积极推行廉政风险防范管理，树立了为民、务实、清廉的良好形象。

各位代表！回顾过去四年的发展历程，政府工作最鲜明的主题是推动科学发展，最突出的主线是转方式调结构，最根本的着力点是惠民生促和谐，最显著的特点是求真务实。在中共淄博市委的坚强领导下，全市上下团结一心，奋发有为，经受住了国际金融危机的严重冲击和国内外复杂环境的严峻考验，出色完成了支援北川灾后重建和“4·28”救援处置等重大急难险重任务，成功举办了第22届省运会等一系列重大赛事，努力开创了殷实和谐经济文化强市建设新局面，充分展示了淄博人民攻坚克难的智慧与勇气、顾全大局的境界与风貌、奋勇争先的干劲与魄力！成绩的取得，得益于党中央、国务院和省委、省政府的科学决策和亲切关怀，胡锦涛总书记、温家宝总理、李克强副总理等中央领导同志亲临我市视察，给全市人民极大鼓舞和有力鞭策。省委、省政府高度关注淄博的发展，对我市工作提出明确要求。成绩的取得，得益于市委的正确领导，得益于市人大、市政协和社会各界的大力支持，得益于全市人民励精图治、艰苦奋斗。在此，我代表市人民政府，向全市人民，向各民主党派、工商联、各人民团体和社会各界人士，向驻淄部队、武警官兵、政法干警和中央、省驻淄单位，向海内外所有关心支持淄博发展的同志们、朋友们，表示衷心的感谢和崇高的敬意！

在充分肯定成绩的同时，我们也清醒地看到，我市经济社会发展还面临一些困难和问题，主要是：经济结构性矛盾依然比较突出，已经成为节能减排、安全生产和生态建设的主要制约因素，可持续发展面临的资源环境和发展空间约束增强；区域创新能力还不够强，高层次和高技能人才相对不足，城市竞争力有待进一步提高；保障和改善民生任务繁重，统筹城乡和区域协调发展、加强和创新社会管理面临许多新课题；面对新形势新要求，政府职能还需要进一步转变，行政服务效能还需要进一步提高。对这些困难和问题，我们高度重视，要采取扎实有效的措施，认真加以解决。

二、认真组织实施好“十二五”规划

市十三届人大四次会议审议通过了《淄博市国民经济和社会发展第十二个五年规划纲要》，经过全市上下一年来的共同努力，我们实现了“十二五”时期的良好开局，站在了新的发展起点上。刚刚闭幕的市十一次党代会确定了今后五年的奋斗目标和主要任务。当前及今后一个时期，我们要认真贯彻落实市十一次党代会精神，进一步深入扎实、与时俱进地组织实施好“十二五”规划，努力抓住新机遇，迎接新挑战，实现新跨越。

按照市十一次党代会对今后五年工作的总体部署和要求，今后一个时期，我们要高举中国特色社会主义伟大旗帜，以邓小平理论和“三个代表”重要思想为指导，以科学发展为主题，以加快转变经济发展方式为主线，紧紧围绕建设殷实和谐经济文化强市的目标，强化生态文明，加快内涵发展，突出加强结构调整，突出统筹城乡发展，突出改善民生维护稳定，加强人才科技支撑，深化改革开放，不断开创淄博老工业城市科学发展新局面。

市十一次党代会确立了强化生态文明、加快内涵发展、建设殷实和谐经济文化强市的总体要求，这是现阶段推动淄博老工业城市科学发展的必然要求和现实选择。要进一步统一思想、形成共识，以更大力度加以推进，着力培植淄博科学发展的长期竞争优势。

——我们要推动经济发展再上新台阶，使淄博的实力更强、后劲更足。发展是富民强市的基础，是解决所有问题的关键。必须毫不动摇地坚持发展是硬道理的战略思想，抓住用好大有可为的重要战略机遇期，千方百计加快内涵发展。要坚持量的增加以质的提升为前提，努力使资源消耗和污染排放更低、投入产出比更高、单位空间创造的价值财富更多、企业核心竞争力更强、产业支撑城市现代化发展的能力更足。要强化科技与人才支撑，推动经济增长向主要依靠科技进步、劳动者素质提高和管理创新转变，向投资、消费、出口协调拉动转变，努力保持强劲增长动力，实现长期平稳较快发展，加快工业总量由1万亿元到2万亿元的跨越，为全面建成小康社会打下具有决定性意义的基础。到2015年，全市地区生产总值突破5000亿元，年均增长12%左右；地方财政收入突破300亿元，年均增长15%左右，城市综合实力和竞争力显著提升。

——我们要推动转型升级实现新突破，使淄博的经济结构更加优化、竞争优势更加突出。转方式调结构事关全局与长远，刻不容缓，必须更加自觉地在发展中促转变，在转变中谋发展，加速以产业升级带动城市转型。要集中调高调强新材料、精细化工、现代医药、汽车及机电装备、新能源与节能环保装备、电子信息等支柱产业，逐步解决工业结构偏重的问题，加快由工业大市向工业强市跨越，把淄博建设成为名副其实的鲁中工业重镇；集中调大调活现代物流、金融保险、文化旅游等现代服务业，加快建设鲁中商贸中心、鲁中物流“旱码头”、鲁中金融中心和品牌旅游目的地，把淄博建设成为鲁中高端服务业聚集区；集中调精调优蔬菜、畜牧、果业、林木花卉等高效特色产业，加快建设南部生态有机农业区、中部观光休闲农业区、北部精准高效农业区三大板块，构建现代农业产业体系。力争“十二五”末，服务业增加值占GDP的比重提高到43%以上，战略性新兴产业进一步形成规模效应，高新技术产业产值比重提高5个百分点以上，形成淄博经济新的竞争优势。

——我们要开创城乡统筹发展新局面，使淄博的城市品质更加优秀、城乡发展更加协调。当前及今后一个时期，是工业化、城镇化快速推进的关键时期，必须抓住机遇，发挥优势，全面加快新型城镇化和城市现代化进程。要按照“现代化双百城市”目标和大城市标准做大做强做美做畅中心城区，在改造提升老城区的同时，加快推动新城区建设，完善提升文化体育功能区，启动建设金融商务和行政服务功能区，彰显现代都市气韵品质，增强服务带动功能。对大外环以内260平方公里区域进行统筹规划，加快构建生态质量良好、产业布局合理、城市结构科学、聚集辐射有力的科学发展核心区。按照中等城市和小城市标准搞好次中心城区和中心镇建设，提高中心村(农村社区)规划建设和公共服务水平，形成四个层级紧密衔接、梯次辐射、互动发展的现代城镇体系和城乡一体化发展新格局。切实完善统筹城乡的制度设计，努力缩小城乡差距，逐步让广大农民过上和城里人一样的生活。力争“十二五”末，城镇化率提高到68%以上，实现省“十二五”规划确定的把淄博建设成为270万城镇人口的区域性中心城市的目标。

——我们要推动生态文明建设实现新跨越，使淄博的生态更美、环境更优。面对日趋强化的资源环境约束，必须更加自觉地把生态文明理念贯穿到经济社会发展的全过程和各领域，加快构建资源节约、环境友好的生产方式和消费模式，在努力清还生态环境“老账”的同时，坚定地走好绿色低碳环保可持续发展的新路子。到2015年，全市万元GDP能耗降低17%以上，COD、SO2排放总量分别削减13%、20%以上，城市污水集中处理率达到98%，生活垃圾无害化处理率保持在100%，全面完成“十二五”节能减排目标任务。要扎实推进森林围城、清水润城等重大生态建设工程，持续改善水体质量和空气质量，加快建设生态淄博，打造鲁中生态高地。到“十二五”末，新增造林面积50万亩、园林绿地1400公顷，全市森林覆盖率达到38%，建成区绿化覆盖率达到46%，积极创建国家生态园林城市和环保模范城。

——我们要迈出文化强市建设新步伐，使淄博的文化更繁荣、城市更文明。文化软实力是城市综合实力和核心竞争力的重要组成部分，必须深入贯彻党的十七届六中全会精神，全面促进文化大发展大繁荣。要加强社会主义核心价值体系建设，弘扬诚信、务实、开放、创新的城市精神，全面提升全国文明城市建设水平。深入实施文化惠民工程，切实加强重大文化工程和基层文化设施建设，健全公共文化服务体系。要坚持以改革创新为动力，大力推动文化与经济社会融合发展，加快构建现代文化产业体系，尽快把文化产业培育成为国民经济新的支柱性产业，努力实现由文化大市向文化强市的跨越。

——我们要推动民生和社会建设再上新水平，使淄博的社会更和谐、人民更幸福。保障和改善民生是一切工作的出发点和落脚点，必须始终坚持以人为本，进一步调整财政支出结构，加大民生与社会建设力度，不断提高城乡公共服务能力和群众生活水平。要积极稳定和扩大就业，显著提高覆盖城乡全体居民的社会保障水平。实施城乡居民收入倍增计划，实现居民收入增长与经济发展同步、劳动报酬增长与劳动生产率提高同步。到2015年，城镇居民人均可支配收入、农民人均纯收入分别达到3.8万元和1.7万元，人民群众的幸福感和满意度不断提高。要大力加强和创新社会管理，促进社会公平正义，加强诚信体系建设，保持社会和谐稳定，努力把淄博建设成为最安全的城市之一和法治环境最好的地区之一。

各位代表！淄博已经站在一个新的历史起点上。在推动“十二五”发展的征途中，我们必须进一步增强机遇意识和忧患意识，勇于在变化的形势中把握机遇，在激烈的竞争中增创优势。经过几年努力，实现“十二五”规划的各项目标，淄博的综合实力将更加雄厚，竞争优势将更加突出，人民生活将更加殷实，“十三五”发展的基础将更加坚实。我们坚信，有市委的坚强领导，有全市上下的不懈奋斗，“十二五”规划的宏伟蓝图一定会实现！

三、关于2012年经济社会发展的目标任务和工作重点

2012年是实施“十二五”规划承上启下的重要一年，党的十八大将要召开，做好今年政府工作具有十分重要的意义。考虑到与市十一次党代会和“十二五”规划确定的目标相衔接，今年全市经济社会发展的主要预期目标建议为：国内生产总值增长10.5%以上，地方财政收入增长15%以上，固定资产投资增长18%以上，社会消费品零售总额增长16%以上，外贸进出口增长10%左右，实际利用外资增长10%以上，城市居民人均可支配收入和农民人均纯收入均增长10%以上，城镇登记失业率控制在4%以内，完成节能减排年度目标任务。

重点抓好以下四个方面的工作：

(一)全力保持经济平稳较快增长。在内外环境不确定因素增多的情况下，保持平稳较快增长对经济发展全局至关重要。要正确把握国家宏观调控取向，促进投资、消费、出口协调拉动。

保持有效投资稳定增长。一是加快推进重点项目建设。突出抓好中央和省投资在建续建项目，有序推进“十二五”规划重点项目，高水平策划实施一批国家、省重点支持的重大项目。牢固树立服务企业发展以项目为纲的理念，进一步加大工作力度，优化投资结构。二是积极拓宽融资渠道。努力加大信贷投放规模，保障重点项目建设和企业正常生产经营的资金需求。积极扩大直接融资规模，力争实现企业上市融资新突破，支持齐鲁股权托管交易中心完善功能、稳健扩容。认真落实国家和省促进民间投资的政策措施，促进民间投资稳定增长。依法严厉打击非法集资等违法犯罪行为，保障金融稳定运行，有效防范系统性风险。三是用好重大政策利好。研究用好高青县列入黄三角高效生态经济区、沂源县享受国家支持沂蒙革命老区政策、淄川区列入国家资源枯竭型城市等重大政策，找准切入点、结合点，高水平策划项目，高效能推进项目，争取最好的政策效益。

抓住用好扩大消费的历史机遇。把扩消费与增投资更好地结合起来，增强经济增长的内生动力。一是落实扩大消费的政策，惠及城乡群众。着力增加低收入者收入，提高中等收入者比重，增强居民消费能力。培育消费热点，引导房地产业健康发展，扩大市内文化旅游、休闲健身、家政养老等服务消费，促进消费升级。二是研究用好扩大消费需求的大势，拉动地方经济增长。加快构建现代商贸流通体系，加强“万村千乡”市场建设，扩大“农超对接”规模，拓展城乡消费市场。调整产业产品结构，着力培植一批高端生活消费品牌，占领国际国内高端市场。提升陶博会等重点展会影响力，积极帮助企业开拓市场。三是优化消费环境，保持物价总体稳定。加强价格监管，依法查处各类价格违法行为。完善价格调节基金制度，健全社会救助和保障标准与物价上涨挂钩联动机制。健全肉类、蔬菜、酒类等重点商品追溯体系，确保放心消费。

推动开放型经济转型升级。一是优化利用外资结构。突出抓好产业链招商、产权招商和境外上市，把实体经济及城市资源存量优势转化为资本优势。加强对现有外商投资企业的服务，鼓励增资扩股、以外引外。坚持引资引智并举，积极引进我市急需的海外人才与技术。二是转变外贸发展方式。综合用好退税、出口信用保险等政策，支持优势出口企业巩固和扩大国际市场份额。充分发挥淄博保税物流中心的口岸服务功能，加强外贸转型示范基地和科技兴贸基地建设，推动出口企业集聚发展。积极发展服务贸易特别是服务外包产业，培育新的外贸增长点。抓住国家支持进口的机遇，扩大重要能源资源和关键设备进口。三是进一步做好“走出去”的文章。积极推进境外资源开发、富余产能转移和海外工程承包，重点支持宏达矿业等境外投资和资源开发项目，规划建设好石嘴山淄博工业园等一批“飞地园区”，承接我市产业梯次转移。

(二)深入推进经济结构战略性调整。工业总量过万亿，标志着我市经济站在了一个更高的发展平台上。要以更大的决心和力度推进经济结构调整，加速新的转型升级。

推动工业大市向工业强市跨越。一是以重点项目为载体，扎实推进“双轮驱动”。深入实施新一轮技改计划和战略性新兴产业倍增工程，加快改造提升石化、轻工、建材、冶金、机械等传统产业，培植壮大新材料、精细化工、现代医药、汽车和机电装备、新能源与节能环保装备、电子信息等新兴产业。安排重点工业项目102项，总投资509.8亿元，其中新兴产业项目数量及投资均占70%以上。下大气力完善提升产业链，集中突破我市优势产业链上的关键节点和核心技术，着力向高端研发和品牌运营延伸，提高产业核心竞争力。重点支持一批“两化”融合示范项目，搞好省级信息化与工业化融合试验区建设，推进信息化与工业研发生产和经营管理的深度融合。二是以骨干企业为支撑，集中培植壮大保增长、转方式、调结构的中坚力量。进一步把各类优质要素资源向核心竞争力强、内涵发展战略清晰、对地方发展贡献大的强企业、大集团集中，优先支持其上市融资、兼并重组、做大做强。深入开展“品牌建设年”活动，培育一批具备突出市场竞争优势的一流品牌。加强中小企业服务体系建设，认真落实对小微企业的财税和金融等扶持政策。三是以重点园区为依托，提高产业集聚集约发展水平。强化主体功能区划，推动企业向园区集中，解决布局散乱和污染围城、污染扩散问题。加快推进中心城区南部区域改造升级。强化技术含量、投入强度、能评环评、单位产出等指标约束，切实提高淄博高新区、东岳氟硅材料产业园、齐鲁化工区等重点园区以及新材料、生物医药等国家级特色产业基地的建设发展水平，打造聚集国内外先进生产要素、发展优势产业集群的重要平台。

推动服务业提质增效。认真组织实施全市服务业发展“十二五”规划，进一步扩大总量、优化结构、提升效益，力争服务业增加值占比提高2个百分点左右。着力打造四大现代服务业优势品牌。一是打造鲁中商贸中心。加强中心商业区和特色商业街区建设，构建传承城市文脉、彰显城市特色、提升市民生活品质的成熟商圈。支持骨干商贸流通企业实行强强联合和跨行业、跨地区经营，培植一批在全省、全国有重要辐射力的商贸流通“航母”企业。改造提升化工、建材、机电以及纺织服装、家具等大型专业批发市场，提高市场影响力。二是打造鲁中物流“旱码头”。全面提升淄博保税物流中心的辐射带动功能，加快规划建设为保税物流中心配套的出口加工区，努力向综合保税区过渡和发展，打造名副其实的鲁中“无水港”。继续抓好齐鲁化工物流园、鲁中公铁物流园、鲁中商贸物流园等总投资150亿元的一批重点物流项目建设，构筑现代物流产业发展体系。三是打造鲁中区域性金融中心。结合提升新区功能，加快规划建设淄博金融集聚区。按照建设山东股权托管交易中心的市场定位，着力提升齐鲁股权托管交易中心的辐射带动功能。继续引进外埠优质金融机构，加大对优质融资性担保机构的扶持力度，支持地方金融企业做大做强。四是打造国内知名旅游目的地城市。加快推进文昌湖旅游度假区、淄博太空港航天科技文化城等一批重大项目建设，培育旅游发展新亮点，提升“齐风陶韵·生态淄博”品牌影响力。中心城区在现代服务业发展上要率先突破，加快建设高端服务业聚集区，带动经济结构和城市功能加速升级。全面落实国家和省、市支持服务业发展的政策措施，优化服务业发展环境。进一步扩大服务业对外开放，积极引进国内外优质资本和著名品牌，增强服务业上规模、上水平的动力。

提高现代农业发展水平。一是抓好粮食生产和高效特色农业发展。大力开展粮食高产创建活动，突出抓好百万亩优质专用粮基地建设，努力实现粮食生产十连增。深入实施蔬菜、畜牧、果业、林木花卉四大产业振兴计划，整建制推进南部山区有机农产品生产区建设，以10万头山东黑牛产业化项目为依托，加快建设高青黄河三角洲高效生态畜牧区。进一步搞好都市农业示范园区建设，提高农业综合效益。二是大力加强水利基本建设。加快推进南水北调地方配套工程，确保与干线工程同步建成、同步见效。加强小农水重点县和大中型灌区续建配套与节水改造工程建设，完成列入国家和省规划的淄河、孝妇河、北支新河等3条河流治理任务，申报实施一批大中型病险水闸除险加固项目，规划建设一批旱能浇、涝能排的高标准农田。完善引太入张工程，推进“清水润城”及生态水系建设。深入推进农村饮水安全工程，年内再解决12万农村群众的饮水安全问题。三是强化农业科技支撑能力。进一步增加投入，加强基层农技推广、农产品质量监管、重大动植物疫病防控等公共服务机构建设，健全完善现代农业产业技术体系，提高设施农业、精准农业发展水平。搞好农村科普和农民培训工作，不断提高农民的科技素质和科技应用能力。四是增强农业和农村发展活力。全面落实强农惠农富农政策，确保各项支农补贴及时到位。完善扶贫开发政策，科学制定并认真组织实施新十年扶贫开发规划。大力培育农业龙头企业和农民专业合作经济组织，积极稳妥地推进农村土地规模经营。

增强自主创新能力。一是加强技术创新平台建设。积极争取国家技术创新工程试点，支持企业加大技术创新投入，普遍建立技术依托，切实增强创新能力。争取东岳集团获批组建国家重点实验室，加快推进山东省芯片设计研发中心、纺织企业技术创新公共服务平台以及相关优势产业技术创新战略联盟建设。二是积极推进高新技术产业化。深化产学研合作，加大对创新成长型企业的支持力度，在战略性新兴产业领域实施一批重点科技攻关计划项目，争取在国产氯碱离子膜工业应用推广、超级动力电容电池、芯片封装测试及新能源汽车等方面取得突破。精心组织好新材料技术论坛系列活动，拓宽高技术成果引进转化渠道。三是优化区域创新环境。支持高新区加快建设国家级创新型科技园区，增强对区域创新的辐射带动功能。推进企业管理创新，总结推广一批优秀创新成果。坚持人才优先、以用为本，突出抓好高层次领军人才和创新型团队的培养引进，为科技创新提供有力的人才智力支持。

下更大气力推进节能减排和环境保护。认真组织实施“十二五”节能减排综合性工作方案，确保完成节能减排年度目标。一是加大节能减排工作力度。把结构调整和技术进步作为实现节能减排目标的根本性举措，大力推广应用节能减排新技术、新工艺、新设备，组织实施节能技术改造、节能环保产业倍增、合同能源管理三大工程，开展300家重点用能企业节能低碳行动和能效对标，力争结构调整和技术进步拉动万元GDP能耗降低3个百分点以上。坚持不懈地淘汰落后产能，坚决关停能耗高、污染重、不具备安全生产条件的“土小”企业，确保完成国家和省下达的淘汰任务。大力支持低温余热发电以及煤矸石、粉煤灰和赤泥等工业废物的资源化利用，扶持发展一批循环经济示范企业和园区。推进建筑、交通、公共机构等领域节能减排工作。二是加大环境保护工作力度。深入实施大气污染治理再提高工程，按照新的国家排放标准，对电力、钢铁、水泥、焦化等重点行业治污设施进行改造，搞好重点企业脱硫、脱硝设施建设。积极开展企业无尘清洁生产，突出加强南定、果里、凤凰等片区的大气污染治理，加快天然气等清洁能源替代工作，加强机动车排气污染防治。按照省统一部署开展对PM2.5的监测，力争全年城区空气质量良好率达到60%以上。开展新一轮重点流域排污企业限期治理工作，完成4座城镇污水处理厂的新建扩建，城市污水集中处理率达到95%以上，凡直排河道的污水处理厂全部建设生态湿地，确保主要河流水质稳定达到恢复鱼类生长的要求。高度重视做好饮用水源地保护工作，加强重金属和危险废物污染防治，确保环境安全。三是强化土地集约节约利用。落实好全市土地利用总体规划，完善城乡建设用地增减挂钩，扎实推进列入省计划的5个土地综合整治项目。加强节约集约用地绩效考核，强化行业用地定额标准和投资强度控制标准，严肃查处土地违法行为。四是加强节能减排长效机制建设。严格落实能评、环评制度，加强能耗和排污总量控制，适时启动预警调控、阶段性“限批”等有力手段，牢牢掌握节能减排的主动权。强化部门联合执法，坚持对环境违法行为实施“顶格处罚”。把完成五年目标与完成年度目标结合起来，严格目标责任考核，确保任务落实。

(三)着力提高城乡统筹发展水平。坚持城乡一体、统筹发展，协调推进中心城区、次中心城区、中心镇、中心村建设，着力完善现代化城镇体系，建设生态和谐宜居城市。

推进四个层级协调发展。一是进一步增强中心城区辐射带动功能。以建设“现代化双百”城市为目标，按照“一城四区”的功能分区要求，统筹新区建设和旧城改造。扎实推进新城区文化教育设施建设以及行政审批服务中心、医疗中心、商务中心、火车站南广场、华润城市综合体等重点工程，提高完善公用设施配套能力，全面提升中心城区服务和带动功能。二是协调推进次中心城区建设。次中心城区要突出特色、发挥优势，布局实施一批对提升城市形象、完善城市功能有较强带动作用的基础设施、公共服务、生态园林和民生建设项目，进一步形成与中心城区有机衔接、功能互补、融合发展的格局。三是着力加强中心镇村建设。突出抓好马桥、凤凰、昆仑三个试点镇建设，以扩权强镇为动力，在产业发展、城镇建设、生态文明、公共服务、社会管理等方面，充分发挥示范带动效应。进一步优化村庄布局，集中抓好一批中心村或农村社区的基础设施建设和村容村貌整治，提升新农村建设水平。

增强基础设施支撑能力。加快推进晋豫鲁铁路沂源段和寿平铁路建设，完善中心城区大外环附属工程，组织实施北京路南延、省道703张店绕城线路面改造等干线公路建设工程，进一步优化路网结构。切实加强农村公路建设与养护管理，推进城乡客运一体化。落实公交优先发展战略，组织搞好纯电动公交车示范运行，满足市民便捷出行需求。深入开展“电网建设提速活动”，组织实施一批输变电工程，全面推进智能配网建设，确保三年内实现城区智能配网全覆盖。加快新一轮农村电网升级改造，推动新农村电气化建设。尽快实现泰青威天然气管线与沧淄线互通，为产业布局调整、清洁能源置换和产业升级提供有力支持。规划建设淄博环保热电厂，完善城乡垃圾处理体系，进一步巩固城市生活垃圾100%无害化处理成果。

加强城乡生态环境建设。组织实施猪龙河整治改造、四宝山片区生态修复、新城区景观水系建设等一批重点工程，进一步搞好重点区域、重点流域的生态建设。大力推进城乡绿化，加强园林景观、森林围城、荒山绿化、农田林网、“两沿三环”等重点绿化工程建设，年内新增园林绿地300公顷、完成造林12万亩。深入开展生态文明系列创建活动，争取年内50%的镇建成省级生态镇。深入开展城乡环境综合整治，加强对重点区域的脏乱差治理。完善城市管理数字化平台，健全“全时段、全覆盖”的环卫保洁机制，打造整洁优美的人居环境。

(四)大力加强民生和社会建设。全面落实国家和省出台的各项民生政策，不断提高公共服务能力和水平，多办让城乡群众看得见、得实惠的好事实事。

加强保障性安居工程建设。按照省政府下达我市的计划，分解任务，落实项目，年内开工建设各类保障性住房和棚户区改造住房18800套，年底前连同结转项目竣工6800套。进一步加强资金和土地保障，落实好保障性住房配建政策。加大以奖代补力度，充分调动企业参与保障性安居工程建设的积极性。全面推行保障性安居工程质量终身责任制。认真贯彻廉租房、经适房、公租房等三个《管理办法》，确保分配公平、管理规范、群众满意。

做好就业和社保工作。认真落实各项就业和创业政策，多渠道扩大就业，力争年内新增城镇就业7.6万人，新增农村劳动力转移就业6.2万人。突出做好高校毕业生就业工作，深入开展校企合作，充分发挥市人力资源市场和大学生创业孵化中心对就业的促进作用。完善城镇基本养老保险制度，全面解决未参保城镇集体企业退休人员养老保障问题。进一步提高企业退休人员养老金水平，适时提高失业保险金标准和工伤保险待遇水平，提高城乡低保标准与补助水平。

提高社会事业发展水平。坚持教育优先发展，全市财政教育支出占财政支出的比重保持在20%以上。加快推进中小学校舍安全工程和办学条件标准化建设，改善城乡办学条件，促进城乡义务教育均衡发展。深入实施学前教育三年行动计划，全面完成年度幼儿园改扩建任务。高度重视并依法加强校车安全和校园治安工作。完成市属高校银行债务化解任务。深化医药卫生体制改革，把村卫生室和非政府办基层医疗卫生机构逐步纳入基本药物制度实施范围，落实对乡村医生的补偿政策，加大全科医生招募和培养力度，提高基层医疗保健水平。将公立医院改革扩大到三个区县。将政府对新农合和城镇居民医保补助标准提高到每人每年240元。进一步加强计划生育工作，稳定低生育水平。组织承办好2012年体操世界杯淄博站比赛。促进妇女儿童、老龄、社会福利、慈善、残疾人、红十字等各项事业科学发展。积极稳妥地推进事业单位分类改革，增强社会事业发展活力。

大力发展文化事业和文化产业。促进文化事业全面繁荣和文化产业快速发展，加快建设文化强市。加大对公共文化建设的投入，加快市文化中心和区县文化中心建设，提高乡镇综合文化站和村文化大院建设水平。实施文化惠民工程，做好参加第十届中国艺术节的相关准备工作。深化文化体制改革，进一步完善扶持文化产业发展的政策体系。重点培育现代印刷发行、工艺美术文博、创意会展等优势文化产业基地，扶持一批文化龙头企业，加快把文化产业培植发展成为新的支柱性产业。

加强和创新社会管理。进一步整合社会管理资源，推行“六小警务”经验，夯实基层基础工作，提高社会管理和服务水平。坚持以群众工作统揽信访工作，认真排查调处影响发展稳定的突出矛盾和问题，努力把各种不稳定因素化解在基层和萌芽状态。深入实施固本强基维稳工程，加强社会治安综合治理，着力解决好流动人口、特殊人群、网络虚拟社会和社会组织的管理服务问题。毫不放松地抓好安全生产，加强食品药品安全监管，健全应急管理机制，确保社会大局和谐稳定。

四、加强政府自身建设

当前及今后一个时期，是我们应对复杂环境考验、加速新的转型发展的关键时期。各级政府必须切实加强自身建设，转变政府职能，创新管理方式，提高行政效能。

(一)转变政府职能，提高行政效率。政府行政效率的高低，事关企业经营发展和群众生产生活，直接影响城市竞争力。要加快政府职能转变，切实在加强服务中改进管理，努力把淄博建设成为全省行政效率最高、行政收费最低的行政区域之一。突出搞好行政审批“两集中、两到位”改革，从今年3月起，各部门行政许可科要全部进驻市行政服务中心，确保行政许可事项进驻中心到位、行政审批授权到位，从体制上为高效审批、阳光审批奠定基础。加快推进建设项目“联合审批”和“一费制”改革，压缩审批时限，降低行政成本。大力加强电子政务建设，健全完善行政电子监察平台，以信息技术的广泛运用提高行政管理效能。

(二)加强制度建设，推进依法行政。依法行政是政府治理水平的重要标志，政府的所有行为都要于法有据、程序正当。要健全行政决策规则，加强重大决策跟踪反馈和责任追究，提高决策的民主化、科学化水平，特别要更加注重公共安全、社会管理、公共服务领域的政府立法，依靠法治手段加强与创新社会管理。深化综合执法改革，细化、量化自由裁量权，健全执法责任过错追究机制，切实做到规范、公正、文明执法。自觉接受人大及其常委会的监督，主动接受政协的民主监督，认真听取各民主党派、工商联、无党派人士和各人民团体的意见，高度重视司法监督、舆论监督和社会公众监督，切实加强政府内部层级监督和审计、监察等专门监督。

(三)坚持从严治政，改进工作作风。政府工作绩效如何，关键在人。要坚持严字当头，加强对公务员的教育和管理，严明责任，严肃纪律，加大行政问责力度，提高执行力，确保令行禁止、政令畅通。全体政府工作人员特别是各级领导干部，要勇于担当，敢于负责，始终坚持发展、发展、再发展，务实、务实、再务实，以务实勤勉、真抓实干的工作，创造真正经得起实践、群众、历史检验的业绩。要密切联系群众，问政于民、问需于民、问计于民，多办顺民意、解民忧、暖民心的实事。大力精简会议和文件，继续压减“三公”支出，把有限的资源和财力用在发展经济、改善民生上。要深入推进反腐倡廉制度创新，健全和落实“一岗双责”，全面推行廉政风险防范管理，认真执行《廉政准则》，进一步加强纠风专项治理，严肃查处各类违法违纪案件，为科学发展提供坚强保障。

各位代表！奋力推动新的转型发展，切实提高淄博的城市地位和竞争力，不断增进全市人民的福祉，是历史赋予我们的崇高使命。让我们在中共淄博市委的坚强领导下，深入贯彻落实科学发展观，解放思想，励精图治，开拓进取，为建设殷实和谐经济文化强市而努力奋斗！
